# Dendritic Cell-Targeted Approaches to Modulate Immune Dysfunction in the Tumor Microenvironment

**DOI:** 10.3389/fimmu.2013.00436

**Published:** 2013-12-10

**Authors:** Anne Gallois, Nina Bhardwaj

**Affiliations:** ^1^Division of Hematology and Oncology, Tisch Cancer Institute, Icahn School of Medicine at Mount Sinai, New York, NY, USA

**Keywords:** cancer, immunotherapy, dendritic cells, tumor microenvironment, immune checkpoints

## Abstract

There has been enormous progress this past decade in the understanding of the biology of dendritic cells (DCs) along with increasing attention for the development of novel dendritic cell (DC)-based cancer therapies. However, the clinical impact of DC-based vaccines remains to be established. This limited success could be explained by suboptimal conditions for generating potent immunostimulatory DCs as well as immune suppression mediated by the tumor microenvironment (TME). Therefore, strategies that optimize the potency of DC vaccines along with newly described therapies that target the TME in order to overcome immune dysfunction may provide durable tumor-specific immunity. These novel interventions hold the most promise for successful cancer immunotherapies.

## Introduction

Naturally occurring anti-tumor immune responses in cancer patients and in murine tumor models are commonly impaired. Tumor escape as a result of immuno-editing or through local effects of the tumor microenvironment (TME) disables many components of the immune response and ultimately limits the success of immunotherapy. Suppression or modulation of tumor-associated dendritic cell (DC) function by the TME is thought to play a major role in impairing the development of potent anti-tumor immune responses and promoting tumor progression. This review provides an overview of the mechanisms by which the tumor cells and tumor-associated cells co-opt many endogenous host factors and physiological pathways in order to impair immunogenic DC function. An updated overview of DC-based tumor immunotherapies and strategies to target the TME in order to overcome DC dysfunction and treat cancer patients will be discussed. Understanding the underlying mechanisms involved in the modulation of DC-based anti-tumor immunity by the TME will provide opportunities for improving the efficacy of cancer immune therapies.

### Dendritic cell biology

The 2011 Nobel Prize in Medicine or Physiology was awarded to Ralph Steinman for his discovery of dendritic cells (DCs) and their role in adaptive immunity. DCs are the most potent professional antigen-presenting cells (APCs), able to activate adaptive immunity through their capacity to sample the environment and capture, process, and present antigens to T cells (1). Immature DCs in peripheral tissues can capture antigens but due to absence of co-stimulatory molecules, antigen presentation results in induction of tolerance through T-cell deletion, anergy and induction of regulatory, or suppressor T cells. Exposure to pathogens, however, engages the process of maturation which guarantees a well-controlled and targeted immune response.

While maturing, DCs lose their ability to capture antigen, and acquire new features such as enhanced antigen processing and presentation (through upregulation of surface MHC-II molecules); enhanced ability to migrate (through upregulation of the chemokine receptor CCR7); and increased capacity to stimulate T and B cells through cytokine secretion and co-stimulatory molecules. DCs uptake antigens through different mechanisms (phagocytosis, macropinocytosis, and endocytosis) and process them into peptides that are loaded on MHC molecules. The peptide/MHC complexes are then presented to naïve T cells in the lymphoid tissues. Binding of T cells to the MHC-antigen complex and co-stimulatory molecules on DC surface (CD80, CD86, CD40) results in the activation and subsequent differentiation of T cells into effector cells endowed with unique functions and cytokine profiles, capable of launching an antigen specific response. Extracellular antigens (bacteria, parasites, toxins) are presented onto MHC-II molecules and presented to CD4^+^ T cells whereas intracellular antigens (viral proteins) are presented on MHC-I molecules to CD8^+^ T cells. Importantly, DCs are the only APCs able to present extracellular antigens onto MHC-I molecules to CD8^+^ T cells, a process called cross-presentation that is crucial for anti-tumor immunity, however, not all DC subsets may be capable of efficient cross-presentation, and the degree to which they do may be dependent upon the nature of the antigen and route of delivery. Myeloid DCs (mDCs, also know as classical or conventional DCs) and plasmacytoid DCs (pDCs) are the two main subsets of DCs. mDCs are key players in immune responses against pathogenic organisms and tumors. They differentiate from myeloid progenitors, express CD11c and include the dermal DCs, Langerhans cells, interstitial DCs, and interdigitating DCs. mDCs are found in peripheral tissues, lymphoid organs, and in the blood and secrete large amounts of IL-12 upon activation. IL-12 mediates enhancement of the cytotoxic activity of NK cells and CD8^+^ cytotoxic T lymphocytes, is involved in the differentiation of naive T cells into T_H_1 cells, and stimulates the production of interferon-gamma (IFN-γ) and tumor necrosis factor-alpha (TNF-α) by T and NK cells cells. Blood mDCs includes BDCA1^+^ (CD1c^+^) and BDCA3^+^ (CD141^+^) DCs. Recent studies have identified BDCA3^+^ (CD141^+)^ DCs as the human counterpart of CD8α^+^ murine DCs that share several phenotypic and functional properties such as their expression of TLR3 and their ability to secrete IL-12 and IFN-β. Although BDCA3^+^ DCs are widely thought to crosspresent antigens more efficiently than other DC populations, new findings show that DC populations may be comparably effective at presenting exogenous antigens to CD8^+^ T cells as long as the antigen is delivered to early endocytic compartments (2, 3).

Plasmacytoid DCs are the principal producers of type-I interferons (IFNs) in response to microbial and viral infection. They express CD123, BDCA2, and BDCA4 and are primarily found in blood and lymphoid organs such as the thymus, bone marrow, spleen, tonsils, and lymph nodes under steady state conditions. pDCs infiltrate various type of tumor but their role in anti-tumor immune responses remains to be defined as some reports suggest they can promote tumor growth (4).

Dendritic cell maturation involves the production of cytokines that play a role in CD4^+^ T-cell polarization into T_H_1, T_H_2, and T_H_17. Differentiation of T_H_1 cells, key players in immune responses against intracellular pathogens, tumors, and viruses, is driven by IL-12-mediated secretion. Development of T_H_2 cells, involved in responses against parasites (but detrimental in the setting of anti-tumor responses), is though to be induced by the lack of IL-12 as well as by IL-4, thymic stromal lymphopoietin (TSLP), and Matrix metalloproteinase 2 (MMP-2). TGF-β, IL-1β, IL-6, and IL-23 have been implicated in T_H_17 polarization. DCs can also induce naïve CD4^+^ T cells to differentiate into T follicular helper cells whose function is to help B cells to differentiate into antibody-secreting cells, as well as into regulatory T cells which function is to suppress immune responses. DCs also play a role in CD8^+^ T-cell differentiation into effector cytotoxic T lymphocytes. In addition to their ability to mediate adaptive immunity, DCs activate innate immune responses, such as NK cells’ cytotoxicity and cytokine production trough their secretion of IL-12, IL-18, and type I-IFN. DCs also activate γδ T cells, another essential component of the anti-tumor immune response. Finally, DCs are also thought to play a role in the induction of effector memory T cells (TEM) that differentiate into central memory T-cell (TCM), but the mechanisms involved are still unclear. Altogether, these findings make DCs the ideal candidate for cancer immunotherapy as they activate overall immune responses.

Interestingly, it has been shown recently that in early stages of tumor progression, DCs are immunocompetent and able to induce the expansion of specific T-cell responses, whereas DCs in advanced tumors become immunosuppressive (5). Understanding the underlying mechanisms involved in the modulation of DC-based anti-tumor immunity by the TME will provide opportunities for improving the efficacy of immune therapies.

### Tumor microenvironment: A hot bed of immuno-suppressive activity

**Figure 1:**
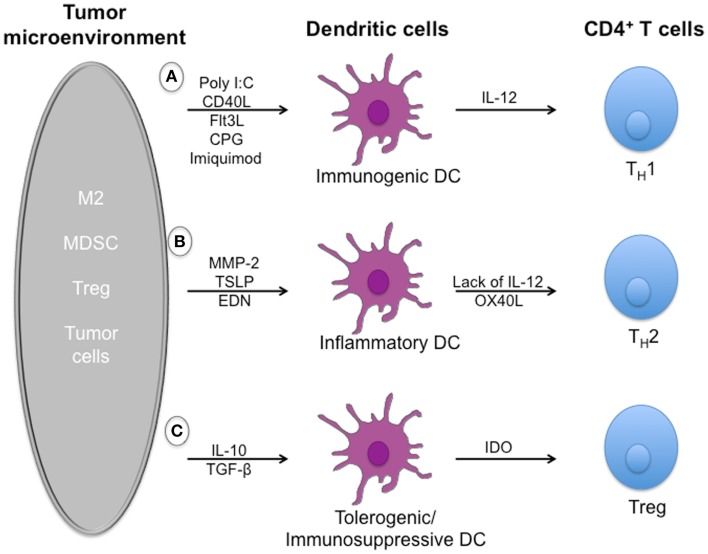
**Dysregulation of dendritic cell-mediated anti-tumor immune responses by tumor microenvironment**. Effector T cells can recognize and kill tumor targets after activation by immunogenic dendritic cells. However, a number of soluble mediators, including TGFβ, IL-10, and alarmins, that are secreted by immuno-suppressive cells such as Treg cells, MDSCs, and tumor cells can dysregulate dendritic cells function and limit T-cell effector functions. **(A)** Exposure to pathogens induces the maturation of immunogenic dendritic cells that secrete large amounts of IL-12 upon activation. IL-12 mediates enhancement of the cytotoxic activity of NK cells and CD8^+^ cytotoxic T lymphocytes, is involved in the differentiation of naive T cells into T_H_1 cells, and stimulates the production of interferon-gamma (IFN-γ) and tumor necrosis factor-alpha (TNF-α) from T and NK cells cells. **(B)** In the tumor microenvironment, development of detrimental/suboptimal T_H_2 cells is induced by alarmins such as TSLP, EDN, and MMP-2 through mechanisms depending on inflammatory DCs. **(C)** Immuno-suppressive cytokines such as IL-10 and TGF-β are responsible for the induction of immature/tolerogenic/immuno-suppressive DCs able to promote the accumulation of regulatory T cells. Tregs play a crucial role in maintaining a suppressive environment and inhibiting anti-tumor responses.

Despite the induction of tumor-specific T-cell responses in many patients, DC vaccines have not translated into durable therapeutic responses. Indeed, the TME employs several mechanisms that inhibit DCs to induce efficient anti-tumor responses (Figure 1).

#### Immuno-suppressive molecules

Several tumor-derived factors such as IDO/TDO, CCL-2, VEGF, TGF-β, M-CSF, GM-CSF, IL-6, and IL-10 have been reported to negatively impact DC functions. TGF-β results in impairment of DC function and accumulation/differentiation of Tregs, myeloid-derived suppressor cells (MDSC), and detrimental M2 macrophages (6). IL-6 and M-CSF switch differentiation from monocytes to macrophages rather than DCs (7). IL-10 is able to convert immunostimulatory DCs into tolerogenic APCs and induce anergic cytotoxic CD8^+^ T cells (8). We and others found that inhibition of MAPK pathway in human BRAF^V600E^ mutant melanoma lines reduced production of immuno-suppressive cytokines (IL-6, IL-10, VEGF) and restored IL-12 and TNF-α production by DCs (9, 10). Stat3 is another signaling pathway that has emerged as a critical regulator of immuno-suppressive cytokines. An excellent review discusses various signaling pathways activated in cancers such as Stats, MAPK, and β-catenin (11). The chemokine CCL2 recruits inflammatory monocytes which express its receptor CCR2, as well as metastasis-associated macrophages, therefore promoting malignancy (12). VEGF is involved in several mechanisms of tumor pathophysiology such as inhibition of DC differentiation (13). Several monoclonal antibodies have been developed against VEGF or its receptor in order to prevent angiogenesis and have shown clinical benefits in various cancers. Activation of antigen-specific-Tregs for potent suppressor activity has been shown to be achieved by pDCs and cDC through secretion of the enzyme indoleamine 2,3-dioxygenase (IDO) (14, 15).

#### Regulatory T cells

CD4^+^ CD25^+^ Foxp3^+^ Tregs play a crucial role in maintaining a suppressive environment and inhibiting anti-tumor responses. Tregs express the inhibitory receptors CTLA-4, PD-1, and Tim-3 which contribute to their suppressive function through different mechanisms (16). Some studies indicate that Tregs through CTLA-4 can induce the down regulation of the co-stimulatory molecules CD80 and CD86 on DCs (17). Moreover, Tregs compete for the cytokine IL-2 with other immune cells through their expression of its receptor CD25 with a 100-fold higher affinity (18). Similar mechanisms might apply for other cytokines such as IL-7, IL-15, and IL-12. Finally, Tregs can secrete two of the main immuno-suppressive cytokines: IL-10 and TGF-β that blunt anti-tumor effector cells such as CD4^+^, CD8^+^, and NK.

#### Immuno-suppressive myeloid cells

It is well established that subpopulations of myeloid cells are critical mediators of tumor initiation, angiogenesis and metastasis and are able to inhibit anti-tumor immune responses through a variety of mechanisms. MDSCs for instance play a crucial role in immune evasion within tumors through several immuno-suppressive mechanisms that blunt effector T-cell responses (19). They suppress CD8^+^ T-cell anti-tumor immunity (20, 21) and induce the differentiation of Tregs (22). Not only do they secrete immuno-suppressive cytokines such as IL-10 but also express high levels of NOS (nitric oxide synthase) involved in T-cell apoptosis (19, 23), and Arginase-1 which impair the local proliferative capacity of T cells (24). Macrophages have also been shown to facilitate tumor growth. In the context of TME, macrophages are skewed toward an M2-altered functional phenotype able to produce lower levels of pro-inflammatory cytokines (IL-1β, TNF-α, IL-12) and higher levels of immuno-suppressive cytokines such as IL-10, TGF-β, and VEGF (25–27). Immunotherapeutic approaches aimed at skewing detrimental M2 macrophages into an immuno-competent M1 phenotype may promote effective anti-tumor immunity.

#### Induction of T_H_2 cells through the expression of alarmins

Alarmins are naturally occurring endogenous mediators, rapidly released in response to infection and/or tissue injury by several cell types. These “danger signals” function to alert the host immune system of cell and tissue trauma through activation and recruitment of effector cells of innate and adaptive immunity (28). DCs are able to sense alarmins present in the TME through surface and intracellular receptors.

Matrix metalloproteinase 2 is expressed by cancer and/or stromal cells and is associated with later tumor stages, increased dissemination, and poorer prognosis/survival (29, 30). We have shown that MMP-2 can directly modulate innate and adaptive immune responses toward melanoma by not only being recognized by specific CD4^+^ and CD8^+^ tumor-infiltrating T cells, but also by modulating DC function to polarize T_H_2 responses. We recently identified two pathways whereby MMP-2 functions as a human endogenous “conditioner” that skews CD4^+^ T cells toward a detrimental T_H_2 phenotype. MMP-2 degrades the type I IFN receptor (IFNAR1), thereby preventing STAT1 phosphorylation necessary for IL-12 production (31). Furthermore, we identified that MMP-2 is a direct ligand for TLR2 on DCs, and found that their interaction leads to OX40L up-regulation and T_H_2 skewing (Godefroy et al., in revision).

Thymic Stromal Lymphopoietin has also been described to modulate DC function and drive T_H_2 responses (32). TSLP produced by tumor cells has been shown to induce detrimental T_H_2 cells responsible for increasing tumor growth in breast cancer and pancreatic cancer through the secretion of IL-13 and IL-4 (33, 34).

These findings support the idea that blocking antibodies for MMP-2/TLR2 or TSLP/TSLPR interactions represent a promising strategy for cancer therapy through their ability to polarize type-1 immune responses.

Another alarmin, Eosinophil-derived neurotoxin (EDN) has been shown to activate the TLR2–MyD88 signal pathway in DCs and enhances T_H_2 immune responses (35).

#### Inhibition of antigen presentation by alteration of MHC molecules and loss of tumor antigen expression

The TME alters the ability of DCs to effectively present antigen due to a down regulation or loss of MHC molecules and genes associated with antigen presentation such as transporter associated with antigen processing (TAP), low-molecular-weight protein (LMP), and β2-microtubulin (36). Another mechanism of tumor escape is the loss of tumor-associated antigens (TAA): the natural selection of tumor subclones poorly recognized by the immune system which can thereby survive immune pressure (37).

#### Expression of inhibitory ligands

Immune checkpoints such as CTLA-4, PD-1, Tim-3, LAG3, ICOSL, GITRl, and B7H3 are inhibitory receptors that regulate immune responses to insure tolerance and prevent auto-immune diseases. They will be discussed in section “Therapies Targeting TME.” CD47, a ligand for SIRPα, is a “don’t eat me” signal for phagocytic cells, whose function is to block phagocytosis. CD47 overexpression by human solid tumor cells represents another mechanism of tumor escape by preventing tumor cells to be phagocytosed and eliminated (38). Recent data has shown that its blockade by neutralizing antibodies inhibits migration and metastasis in a variety of tumor models.

Study of the TME is critical to better understand how tumors harness surrounding cells to escape immunity and support their growth. This combined with a better understanding of DC biology should lead to the development of new strategies that effectively restore DC activity and induce tumor detection and the generation of potent anti-tumor responses.

## Dendritic Cell-Based Tumor Immunotherapies

**Figure 2:**
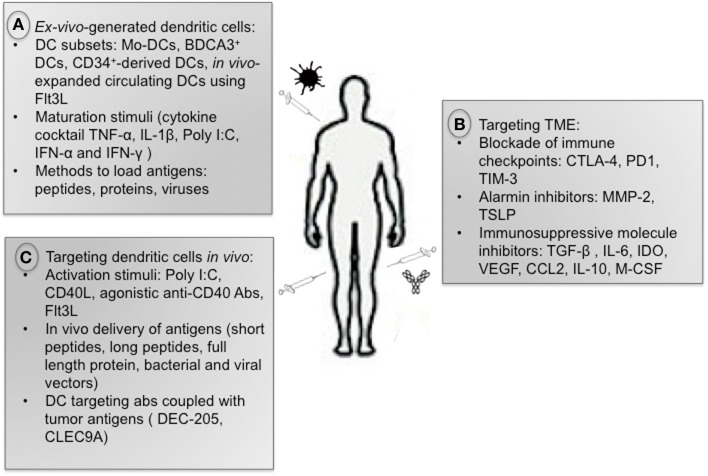
**Anti-tumor immunotherapies**. **(A)** There is currently no consensus on the optimal strategy to generate DCs for immunotherapeutic use regarding DC subsets, maturation stimuli, and methods to load antigens. **(B)** Therapies aiming at reprograming the immuno-suppressive TME are very promising, including blockade of immune checkpoints as well as inhibitors of alarmins and immuno- suppressive cytokines. **(C)** Strategies targeting DCs *in vivo* include administration of activation stimuli (Poly I:C, CD40L, Flt3L), *in vivo* delivery of tumor antigens, and administration of tumor antigens coupled with antibody against DC surface receptors.

The immune system can eradicate tumors as shown by spontaneous regression of primary and metastatic melanoma (39) and regression of tumors after adoptive transfer of T cells (40). The potential for DCs to launch adaptive immunity makes them ideal candidates for cancer immunotherapy (Figure 2). However this approach alone does not overcome TME-induced DC dysregulation. Therefore, targeting TME may improve the clinical benefit of DC-based vaccines.

### DC-based vaccines

#### *Ex vivo*-generated DCs pulsed with antigens

The clinical impact of DC immunotherapy has been limited despite the induction of tumor-specific T-cell responses in many patients and occasional tumor regressions. At this point, the first and only cell-based cancer vaccine approved by the FDA is Provenge^®^ from Dendreon. Provenge is an autologous antigen-pulsed DC-based cancer vaccine for patients with metastatic prostate cancer based on the results of a phase III randomized trial that demonstrated a more than 4-month median improvement in overall survival compared with a placebo vaccine. Overall, clinical trials have demonstrated the feasibility and safety of DC vaccines in phase I and II but have failed to demonstrate strong efficacy in large phase III trials (41, 42).

Many reasons may explain this lack of success with DC vaccines. There is currently no consensus on the optimal strategy to generate DCs for immunotherapeutic use. DC-based immunotherapies require optimization at several levels: the maturation stimuli used, the type and form of antigen to be administered, the subset and the number of DCs to inject, and the frequency, route, and site of the injection. Studies in humans and mice have emphasized that different DC subsets are endowed with specialized functions, and a good vaccine should utilize these subsets in a coordinated way. Questions remain as to whether the classical *ex vivo*-generated moDCs widely used in immunotherapy are the most effective means of inducing clinically significant anti-tumor immunity. Some studies use DCs derived from CD34^+^ precursors (43) or *in vivo*-expanded circulating DCs using Flt3L. Recent findings provide the basis for a new approach relying on BDCA3^+^ DCs as anti-tumor vaccines, as they seem to be a key subset for cross-presentation of cell-associated antigens (44). Further characterization of these DCs will enable rational approaches to target them to improve vaccine efficacy. Looking forward, the main challenge for using BDCA3^+^ DCs will be to develop an efficient way to generate them in large numbers. Alternative vaccination strategies such as the delivery of tumor antigens *in vivo* to BDCA3^+^ DC subsets using antibodies specific to cell surface receptors such as CLEC9A has been proposed. However, more recent findings previously discussed (2, 3) suggest that this approach may not offer an inherent advantage and that the optimal strategy would be to target antigens to early endosomes. This approach would not only increase cross-presentation by BDCA3**^+^** DCs but also extend cross-presentation to more abundant DC subsets therefore maximizing CD8^+^ T-cell responses *in vivo*. It is worth pointing out that Dendreon uses circulating blood DCs as the adjuvant, not the commonly used moDCs. A large study directly comparing all DC subsets side by side for their capacity to induce CTL and T_H_1 responses after activation with various stimuli is warranted.

Another critical parameter to induce DC-mediated potent anti-tumor responses is the choice of DC maturation stimuli. Indeed, proper DC maturation prior to vaccination is necessary to prevent induction of tolerance through Tregs. To mature DCs, some clinical trials have used a standardized cocktail of pro-inflammatory cytokines composed of TNF-α, IL-1β, IL-6, and PGE2 that was shown to induce up-regulation of MHC molecules, co-stimulatory molecules as well as CCR7 (45). However, other findings have suggested that DC matured with this cytokine cocktail were not optimal as they fail to induce IL-12p70 production and may induce Treg and T_H_2 cells (46–48). A novel cytokine cocktail consisting of TNF-α, IL-1β, Poly I:C, IFN-α, and IFNγ has shown good results including DC-mediated IL-12 secretion (49, 50). Alternative maturation strategies *via* direct administration of immune activators such as TLR agonists, Flt3L, or CD40L has been shown to improve DC function *in vivo* (51). Several TLR ligands are currently being tested in clinical trials including LPS (TLR4), CpG (TLR9), Poly I:C (TLR3), Imiqiuimod (TLR7), and Resiquimod (TLR7 and TLR8).

Another factor that may explain the limited success of DC-based vaccines is the less-than optimal migration of DC vaccines to secondary lymphoid organs. Studies showed that most of the injected DCs remain at the site of injection, <5% reaching the draining lymph nodes (52). Administration of DCs via multiple routes or directly into the lymph nodes may improve DC migration and clinical responses.

Finally, it’s worth mentioning that most of the clinical trials treat patients with late stage cancers, whereas the most suitable stage for cancer vaccine is likely to be early disease when tumor volume is low.

#### *In vivo* delivery of antigens (non-targeted vaccines)

Contrary to previous assumptions, we showed that DC vaccines have an insignificant role in directly priming CD8^+^ T cells, but instead function primarily as vehicles for transferring antigens to endogenous APCs, which are responsible for the subsequent activation of T cells (53). This finding highlights the need to develop strategies directly targeting endogenous DCs. Moreover, *in vivo* targeting of DCs represents a more economical option for DC immunotherapy as it bypasses the expensive and labor-extensive *ex vivo* DC generation process described previously.

Tumors express several well-characterized antigens that are recognized by the immune system. TAA can be antigens derived from oncogenic viruses (human papilloma virus E6 and E7 proteins), the products of mutations, differentiation antigens (tyrosinase, TRP-1, TRP-2, gp100, Melan A/MART1), overexpressed variants (Her2/neu), or self-antigens specifically upregulated on tumors. Strategies that target antigen presentation on both MHC-I and II molecules are ideal as both CD4^+^ and CD8^+^ T cells are required to launch potent protective anti-tumor immune response. Immunotherapies using short peptides from tumor antigens present limitations because they can only be used in patients with known HLA alleles that present these epitopes in the absence of natural processing. Alternatively, full-length protein vaccines often suffer from lack of consistent CD8^+^ T-cell induction, likely due to inefficient cross-presentation of the exogenous antigen by DCs. In contrast, synthetic long peptides are efficiently presented to both CD4^+^ and CD8^+^ T cells by DCs as well as non-professional APCs (54). The use of bacterial and viral vectors represents another alternative for loading tumor antigens on DCs. Genes encoding TAAs are inserted into the vector while gene encoding virulence of replication factors are deleted out. In some case, the vector may encode for cytokines and co-stimulatory molecules and therefore induce maturation of DCs, thereby bypassing the need for a separate maturation stimuli (55). The disadvantage of the method is that pre-existing immunity against the bacteria or virus vector may reduce their ability to induce immune responses.

#### Antigens coupled with DC surface antigens (*in vivo* targeting of DCs)

Endogenous DCs can be targeted to either deliver tumor-associated-antigens and/or to provide co-stimulatory signals. Candidates for the targeting of DC-specific molecules include Fc receptors, CD40, and C-type lectin receptors such as DEC-205, DC-SIGN, CLEC9A, mannose receptor, and Dectin-1. TAAs can be directly delivered *in vivo* using chimeric proteins composed of an antibody that is specific for the DC receptor fused to a selected antigen or to long peptides. Specific targeting of antigens to DCs *in vivo* has been shown to elicit potent CD4^+^ T-cell responses as well as an enhancement of antibody responses (56–58). CD8^+^ T-cell responses are less efficiently induced, unless boosted in a “prime” fashion such as with pox vectors (59). To avoid the induction of antigen-specific tolerance, this strategy requires DC activation signals. Most of the studies are performed in mice and further investigations are needed to determine the efficacy in humans and to identify the best candidate to target.

Optimizing DC vaccines is necessary but to be successful, immunotherapeutic approaches also need to overcome TME-induced immune suppression to be able to potentiate the efficacy of DC vaccines *in vivo* and translate to overall improved clinical outcomes.

### Therapies targeting TME

Among the most promising approaches to activating therapeutic anti-tumor immunity is the blockade of immune checkpoints. Among checkpoint molecules, CTLA-4 blockade was the first shown to enhance anti-tumor responses (58). CTLA-4 is an homolog of CD28 whose binding to its ligands CD80 and CD86 induces an inhibitory signals to CTLA-4-expressing T cells. CTLA-4 blockade using neutralizing antibodies (Ipilimumab and Tremelimumab) targets both effector and regulatory Tregs and has been shown to enhance immune responses and show promising clinical responses in melanoma patients (60). Ipilimumab (Yervoy) has recently been approved by the FDA for the treatment of unresectable or metastatic melanoma, based on improved overall survival in treated patients (61). Anti-CTLA-4 treatment is currently being tested for other cancers.

PD-L1, a ligand for the exhaustion marker PD-1, is expressed by different TME-infiltrating cell types including DCs. Blockade of PD-L1 induced durable tumor regression and prolonged stabilization of disease in patients with advanced cancers, including non-small-cell lung cancer, melanoma, and renal-cell cancer (62). Moreover, clinical trials using an anti-PD-1 antibody (nivolumab) reported promising results in patients with advanced cancer (63). Nivolumab is now in phase III testing. Interestingly, early results presented at the ASCO 2013 meeting suggested higher response rates to PD-1 pathway blockade in patients whose tumors express PD-L1, while combinatorial blockade of CTLA-4 and PD-1 increased anti-tumor immunity when compared to blocking either single checkpoint alone, although toxicity was higher (Grosso, abstract #3016; Callahan, abstract #9012).

Similarly to CTLA-4 and PD-1, Tim-3 belongs to the group of immune checkpoints and is a potential therapeutic target. Although there is no clinical data yet, Tim-3 has been reported to be co-expressed with PD-1 on human tumor-specific CD8^+^ T cells, and dual blockade of both molecules significantly enhances the *in vitro* proliferation and cytokine production of human T cells (64–66). *In vivo* studies have shown that Tim-3 blockade alone, or in combination with PD-1 blockade, is able to control tumor growth in four different tumor models, including melanoma (66, 67). Moreover, recent findings have shown that tumor-infiltrating DCs suppress nucleic acid-mediated innate immune responses through interactions between the receptor TIM-3 and the alarmin HMGB1 (68) therefore defining a new mechanism whereby the TME suppresses anti-tumor immunity. We found that NK cells from melanoma patients were dysfunctional/exhausted and that Tim-3 blockade was able to reverse this exhausted phenotype and improve NK cell function. Altogether, those findings suggest that Tim-3 blockade would improve anti-tumor immunity by not only targeting T cells, but also DCs and NK cells.

## Conclusion

Dendritic cells have the potential to initiate specific anti-tumor immune responses, but several components of TME can modify their phenotype and function to transform immuno-competent DCs into immuno-suppressive DCs. The TME not only abrogates specific T-cell response but also induces DCs to exert immuno-suppressive and pro-angiogenic functions. Thus, combinatorial approaches that (1) reprogram the immuno-suppressive TME; (2) improve DC function; and (3) enhance T-cell immunity, should provide durable tumor-specific immunity and hold the most promise for successful immune-base cancer therapies.

## Conflict of Interest Statement

The authors declare that the research was conducted in the absence of any commercial or financial relationships that could be construed as a potential conflict of interest.
